# Novel Mixed Matrix Membranes Based on Poly(vinylidene fluoride): Development, Characterization, Modeling

**DOI:** 10.3390/polym15051222

**Published:** 2023-02-28

**Authors:** Anna Kuzminova, Mariia Dmitrenko, Andrey Zolotarev, Denis Markelov, Andrei Komolkin, Roman Dubovenko, Artem Selyutin, Jiangjiexing Wu, Rongxin Su, Anastasia Penkova

**Affiliations:** 1 St. Petersburg State University, 7/9 Universitetskaya nab., 199034 St. Petersburg, Russia; 2 Sirius University, 1 Olympic Ave, Township Sirius, 354340 Sochi, Russia; 3 Zhejiang Institute of Tianjin University, Ningbo 315201, China; 4 School of Marine Science and Technology, Tianjin University, Tianjin 300072, China; 5 State Key Laboratory of Chemical Engineering, Tianjin Key Laboratory of Membrane Science and Desalination Technology, School of Chemical Engineering and Technology, Tianjin University, Tianjin 300072, China

**Keywords:** poly(vinylidene fluoride), titanium dioxide, graphene oxide, multi-walled nanotubes, pervaporation, ultrafiltration, photocatalytic activity, molecular dynamics simulation

## Abstract

Membrane technology is an actively developing area of modern societies; with the help of high-performance membranes, it is possible to separate various mixtures for many industrial tasks. The objective of this study was to develop novel effective membranes based on poly(vinylidene fluoride) (PVDF) by its modification with various nanoparticles (TiO_2_, Ag-TiO_2_, GO-TiO_2_, and MWCNT/TiO_2_). Two types of membranes have been developed: dense membranes for pervaporation and porous membranes for ultrafiltration. The optimal content of nanoparticles in the PVDF matrix was selected: 0.3 wt% for porous membranes and 0.5 wt% for dense ones. The structural and physicochemical properties of the developed membranes were studied using FTIR spectroscopy, thermogravimetric analysis, scanning electron and atomic force microscopies, and measuring of contact angles. In addition, the molecular dynamics simulation of PVDF and the TiO_2_ system was applied. The transport properties and cleaning ability under ultraviolet irradiation of porous membranes were studied by ultrafiltration of a bovine serum albumin solution. The transport properties of dense membranes were tested in pervaporation separation of a water/isopropanol mixture. It was found that membranes with the optimal transport properties are as follows: the dense membrane modified with 0.5 wt% GO-TiO_2_ and the porous membrane modified with 0.3 wt% MWCNT/TiO_2_ and Ag-TiO_2_.

## 1. Introduction

Currently, water treatment is a global problem of modern society [1]. Various methods are used for water purification, and among the promising methods are membrane processes since they have such qualities as environmental friendliness, cost-effectiveness, low energy consumption, and compact equipment used for their implementation [2]. Effective implementation of water purification using membrane technologies requires new high-performance membrane materials with improved properties and cleaning ability after UV irradiation [3]. Mixed matrix membranes, in which an inorganic and/or organic particle is added to the polymer matrix, are currently in active development, giving new improved characteristics to polymer membranes [4,5]. For the manufacture of mixed matrix membranes, metal-organic frameworks (MOFs) [6], fullerenol [7,8], graphene oxide [9,10], polymers [11], carbon nanotubes [12,13], metal oxides [14,15,16,17,18], etc. have actively been used as modifiers in recent years. Metal oxides are promising modifiers since they not only improve the transport characteristics of membranes but also, due to their photocatalytic properties, give membranes the ability to self-purify in separation processes [19,20]. The most common photocatalyst is titanium dioxide (TiO_2_), as it is a highly efficient and inexpensive photocatalyst [21]. Titanium dioxide is widely used to improve the transport characteristics of polymers such as polyacrylonitrile (PAN) [22], poly(m-phenylene isophtalamide) (PA) [23], polysulfone (PSF) [24,25], polyethersulfone (PES) [26], and poly(vinylidene fluoride) (PVDF) [16,18,27]. Recently, in addition to the use of just titanium oxide, modified particles have become widespread. Titanium oxide is modified using metals [18,28,29,30], graphene oxide [31,32,33,34], metal oxides [35,36], carbon nanotubes [37,38], and their mixtures [39,40]. In this case, most often, membranes with modified titanium oxide in the matrix have improved transport and photocatalytic properties. Therefore, in this study, the influence of both titanium oxide alone and modified titanium oxide particles was studied.

A common polymer, PVDF, was chosen as the polymer matrix. PVDF is composed of repeating units—(CH_2_-CF_2_)_n_—and a semicrystalline polymer. It has found wide applications in membrane technology, as it has good chemical resistance, high mechanical strength, thermal stability, aging resistance, and low cost; therefore, PVDF membranes are suitable for manufacturing on a large scale [41,42]. PVDF-based membranes are used in ultrafiltration [16,18,27,33,43,44,45,46,47], microfiltration [48,49], membrane distillation [50,51,52], and pervaporation [53,54,55]. In this study, ultrafiltration and pervaporation PVDF-based membranes were developed. Most often, researchers obtain porous or asymmetric membranes based on PVDF since this polymer has a dense structure and low permeability. Therefore, these membranes are mainly used in ultrafiltration. PVDF-based membranes are used to remove proteins [16,18,33,43,44], dyes [45,46], *E. coli* [18,27], hormones [47], and oil [56,57]. Pervaporation membranes based on PVDF are not as common. Thus, membranes were developed for the separation of organic media [53] and water treatment [54,58]. In these works, not only were dense membranes developed but also asymmetric ones since they have greater productivity. Despite excellent performance, PVDF has poor transport characteristics due to the high hydrophobicity of the polymer. Therefore, increasing hydrophilicity to increase permeability is the main goal [59]. The properties of PVDF-based membranes are affected by various factors, such as solvent composition, solution preparation temperature, polymer concentration, membrane formation method, the presence and concentration of modifiers, etc. [60].

PVDF is one of the most resistant polymeric materials to UV irradiation, and therefore, its modification with photocatalysts is extremely relevant [59]. The novelty of this study consisted in a detailed investigation of the effect of titanium oxide unmodified and modified with nanoparticles (Ag-TiO_2_, GO-TiO_2_, and MWCNT/TiO_2_) on the structure, physicochemical, and transport characteristics of PVDF membranes obtained by two different methods: non-solvent induced phase separation (NIPS) for forming a porous structure of a polymer membrane and the evaporation induced phase inversion method (EIPS) for forming a dense structure of a polymer membrane. It should be noted that, to our knowledge, there are no works on the modification of PVDF by the particles used to obtain mixed matrix pervaporation membranes.

The aim of this study was to investigate the effect of modifiers and the method of membrane formation on the transport, structural, and physicochemical properties of PVDF-based porous and dense (non-porous) membranes. To increase the hydrophilicity and permeability of the PVDF membrane, various nanoparticles (TiO_2_, Ag-TiO_2_, GO-TiO_2_, and MWCNT/TiO_2_) were introduced, and their optimal concentration was found. The structural and physicochemical properties of the developed membranes were studied using different methods of analysis. To study the influence of the modifier on the hydrophilicity and performance of the modified PVDF membrane, the molecular dynamics (MD) simulation of PVDF and the TiO_2_ system was carried out. The transport properties and cleaning ability under ultraviolet irradiation of porous membranes were studied by ultrafiltration of a bovine serum albumin (BSA) solution coolant lubricant suspension (CL). The transport properties of dense membranes were studied in pervaporation separation of a water/isopropanol (50/50 wt%) mixture.

## 2. Materials and Methods

### 2.1. Materials

Poly(vinylidene fluoride) (PVDF, XF2170P, molecular weight of 300,000–500,000 g/mol, Transcool LLC, Moscow, Russia) was used as the membrane material. Titanium dioxide (TiO_2_, ~21 nm, Sigma-Aldrich, St. Louis, MO, USA) and multi-walled nanotubes (MWNTs, Fullerene Technologies, St. Petersburg, Russia) were applied for the modification of poly(vinylidene fluoride). Graphene oxide (GO, Fullerene Technologies, St. Petersburg, Russia) was used for modification of TiO_2_ (GO-TiO_2_). Silver nitrate (AgNO_3_, LenReactive, St. Petersburg, Russia) was used for modification of TiO_2_ (Ag-TiO_2_). Isopropanol (i-PrOH), ammonia (NH_4_OH), N,N′-dimethylacetamide (DMA), and glycerol (Vekton, St. Petersburg, Russia) were used without further purification. Polyvinylpyrrolidone K-30 (PVP, Sigma-Aldrich, St. Petersburg, Russia) was used as a pore former for porous PVDF membranes. Bovine serum albumin (BSA, Mw = 67,000 g/mol, Sigma-Aldrich, St. Louis, MO, USA) was used for ultrafiltration separation as a solution in phosphate buffer (pH = 7.0–7.2, 0.5 wt%) due to its optimal conformation at this pH [61,62,63,64,65,66]. Coolant lubricant (CL, Wittol 297, SERVOVIT, Minsk, Belarus) was used as an emulsion in water for ultrafiltration separation.

### 2.2. Modification of TiO_2_ Particles

#### 2.2.1. Preparation of GO–TiO_2_ Composites

A GO-TiO_2_ composite was synthesized by the one-stage hydrothermal method as follows [33]: the GO powder was added to an ethanol:water mixture (2:1 in volume) and dispersed using ultrasound for 1 h. Next, TiO_2_ powder (GO:TiO_2_—1:9 by weight) was added to the resulting suspension and stirred for 2 h until a homogeneous suspension was formed. Next, the resulting suspension was transferred to an autoclave and kept at a temperature of 120 °C for 3 h. GO-TiO_2_ particles were recovered by centrifugation, washed with deionized water, and dried for 24 h under vacuum at 60 °C.

#### 2.2.2. Preparation of Ag–TiO_2_ Composites

To obtain an Ag-TiO_2_ composite, three solutions were prepared: (1) AgNO_3_ was dissolved in 10 mL of water at a concentration of 40.8 g/L with stirring; then, ammonia was added dropwise to the solution until the precipitate completely disappeared. (2) TiO_2_ was dissolved in 10 mL of ethanol using ultrasound at a concentration of 30 g/L. (3) PVP K-30 was dissolved in 50 mL ethanol at a concentration 20 g/L. Next, solutions 1 and 2 were mixed with constant stirring for 1 h and then mixed with solution 3. The resulting solution was stirred for 7 h at 70 °C. Then, the resulting composite was separated by centrifugation, washed repeatedly with water and ethanol, and dried in vacuum at 60 °C [18].

### 2.3. Porous Membranes Preparation

TiO_2_, Ag-TiO_2_, GO-TiO_2_, and MWCNT with pore former PVP K-30 were introduced into the PVDF matrix by the solid-phase method, which consisted of the following: PVDF powder was ground with the calculated amount of modifier and PVP K-30 relative to the polymer weight in agate mortar with further dissolution of composites in a DMA at 100 °C during 3 h to obtain 15 wt% solution. MWCNT and TiO_2_ were added at PVDF in a proportion of 2:1; in this case, the percentage content in the PVDF matrix was calculated in relation to TiO_2_. The porous PVDF membrane was prepared by the phase inversion technique with non-solvent induced phase separation (NIPS): the PVDF solution at 25 °C was cast with a casting blade at 200 μm onto a glass plate with the following immersion in a coagulation bath with the distilled water.

### 2.4. Dense Membranes Preparation

TiO_2_, Ag-TiO_2_, GO-TiO_2_, and MWCNT were introduced into the PVDF matrix by the solid-phase method, which consisted of the following: PVDF powder was ground with the calculated amount of modifier relative to the polymer weight in agate mortar with further dissolution of composites in a DMA at 25 °C for 5 h to obtain a 10 wt% solution. MWCNT and TiO_2_ were added at PVDF in a proportion of 2:1; in this case, the percentage content in the PVDF matrix was calculated in relation to TiO_2_. The dense PVDF membrane was prepared by the evaporation induced phase inversion method (EIPS): the prepared PVDF solution or composites with nanoparticles was applied to glass to form a polymer film ~30 ± 5 µm thick by evaporation of the solvent for 24 h at 60 °C.

Table 1 shows the designations of dense and porous membranes developed in this study.

### 2.5. Fourier Transform Infrared Spectroscopy

Structural changes in the dense PVDF and PVDF/nanoparticles pervaporation membranes were studied by Fourier transform infrared spectroscopy (FTIR) using spectrometer BRUKER-TENSOR 27 (Bruker, St. Petersburg, Russia) with an attenuated total reflectance (ATR) accessory attached. The measurement was carried out in the range of 600–4000 cm^−1^ at ambient temperature.

### 2.6. Atomic Force Microscopy

The topography of the surface of the dense and porous PVDF and PVDF/nanoparticle membranes was studied by atomic force microscopy (AFM) using the atomic force microscope NT-MDT NTegra Maximus (NT-MDT Spectrum Instruments, Moscow, Russia) with a rigidity of 15 N·m^−1^ in the tapping mode and standard silicon cantilevers. AFM images were obtained as follows: an area of 100 × 100 µm was scanned on the membrane; then, at least five 10 × 10 µm areas were cut from this area in different places, and the roughness data were calculated using the program Nova. The obtained results were averaged.

### 2.7. Scanning Electron Microscopy

The inner and surface morphology of dense and porous PVDF and PVDF/nanoparticle membranes were studied by scanning electron microscopy (SEM) using the scanning electron microscope Zeiss AURIGA Laser (Carl Zeiss SMT, Oberhochen, Germany) at 1 kV. The cross sections of dense and porous membranes were obtained by cooling the membranes in liquid nitrogen, followed by fracturing perpendicular to the surface.

### 2.8. Thermogravimetric Analysis

The thermochemical properties of the dense PVDF and PVDF/nanoparticle membranes were studied by thermogravimetric analysis (TGA) using Thermobalance TG 209 F1 Libra (Netzsch, Leuna, Germany) in an argon atmosphere at a heating rate of 10 °C/min from 30 to 613 °C.

### 2.9. Contact Angle Measurements

Changes in the surface hydrophilic/hydrophobic balance of dense membranes were studied by measuring the contact angles in a static mode using the Goniometer LK-1 instrument (NPK Open Science Ltd., Krasnogorsk, Russia). The DropShape software was used to analyze the results. The sessile drop method was used for dense membranes, which consisted in applying a drop of water or glycerol to the surface of the membrane. A snapshot of the contact angle was taken after 3 s, while the solvent drop was stable for 10 s.

For porous membranes, the contact angles were studied by the attached bubble method, which consisted in immersing the membrane in water and attaching an air bubble to the surface of the membrane [23]. A snapshot of the contact angle was also taken after 3 s, while the air bubble was also stable for 10 s. At least 5 drops of solvent and air bubbles were attached to the surface of the membranes, and the data obtained were averaged.

### 2.10. Critical Surface Tension

To measure critical surface tension *σ_s_*, contact angles were measured for two liquids: water and glycerol. Critical surface tension *σ_s_* was determined by

(1) $$\sigma_{s}=\sigma_{s}^{d}+\sigma_{s}^{p},$$

where *σ_s_* is the critical surface tension,$\;\sigma_{s}^{d}$ is the dispersion component, and $\sigma_{s}^{p}$ is the polar component.

Dispersion and polar components can be calculated in terms of the contact angles according to

(2) $$\cos\left(\theta +1\right)\cdot \sigma_{l}=2\cdot \left(\sqrt{\sigma_{l}^{d}\cdot \sigma_{s}^{d}}+\sqrt{\sigma_{l}^{p}\cdot \sigma_{s}^{p}}\right),$$

where *θ* is the contact angle,$\;\sigma_{l}$ is the surface tension of liquid, $\sigma_{l}^{d}$ and $\sigma_{l}^{p}$ are the dispersion and polar components of liquid, respectively, and $\sigma_{l},\;\sigma_{l}^{d},\;\mathrm{and}\;\sigma_{l}^{p}$ are reference data.

### 2.11. Pervaporation Experiment

The transport properties of the developed dense PVDF and PVDF/nanoparticle membranes were studied at pervaporation using a laboratory cell in a stationary mode at 22 °C [67]. The compositions of the permeate and feed were investigated using a gas chromatograph Chromatec Crystal 5000.2 (Chromatec, Nizhny Novgorod, Russia) with a column “Hayesep R” and a thermal conductivity detector.

The areas of chromatographic peaks were determined by triangulation. Samples of analyzed liquids were injected with a DAZH-2M autosampler (Chromatec, Nizhny Novgorod, Russia) with a volume of 0.2 µL. The chromatograms were processed based on the internal normalization method. Isopropanol was chosen as the standard substance. Mass fractions of substances in mixtures were determined by

(3) $$C_{i}=\frac{f_{i}\cdot P_{i}}{\sum_{i}^{n}f_{i}\cdot P_{i}},$$

where *P_i_* is the chromatographic peak area, *f_i_* is the normalization (calibration) factor, and *n* is the number of substances in the mixture.

Chromatograms were taken 3–5 times. For each of the samples, the composition was calculated according to Equation (3), after which the obtained values were averaged.

To calculate the permeation flux *Q* (kg/(m^2^h)) of the dense PVDF and PVDF/nanoparticle membranes, the following equation was used [68]:

(4) $$Q=\frac{W}{A\cdot t\prime }$$

where *W* (kg) is the weight of the permeate, *A* (9.6 × 10^−4^ m^2^) is the effective membrane area, and *t* (h) is the time of the measurement.

All the data were collected 3 times, and the average value was used. The obtained average accuracies were as follows: ±0.5% for water content in the permeate, and ±5% for permeation flux of the dense PVDF and PVDF/nanoparticle membranes.

### 2.12. Ultrafiltration Experiment

The transport properties of the developed porous PVDF and PVDF/nanoparticle membranes were studied at ultrafiltration using a laboratory cell in a stationary mode at 22 °C [23]. A BSA solution in phosphate buffer (0.5 wt%) at pH = 7 and emulsion of CL (5 wt%) in water were used as the feed for ultrafiltration separation.

To calculate the pure water and feed flux of the porous PVDF and PVDF/nanoparticle membranes, the following equation was used [69]:

(5) $$J=\frac{V}{A\cdot t\prime }$$

where *V* (L) is the permeate volume, *A* (24.6 × 10^−4^ m^2^) is the effective area of the porous membrane, and *t* is the time of the measurement (h).

The content of BSA and CL in the permeate and feed was studied by spectrophotometry using a Spectrophotometer PE-5400UV at 280- and 500-nm wavelengths, respectively. The concentration of BSA and CL in the permeate and the feed was determined from the calibration curve constructed from 0.01 to 0.5 wt% and 0.01–5 wt%, respectively.

To calculate the rejection coefficient of the porous PVDF and PVDF/nanoparticle membranes, the following equation was used:

(6) R=(1−CpCf)×100%,

where *C_p_* and *C_f_* are the content of BSA in the permeate and the feed (wt%), respectively.

To calculate the flux recovery ratio (FRR) of the porous PVDF and PVDF/nanoparticle membranes, the following equation was used [70]:

(7) FRR=(JJ0)×100%,

where *J* is the pure water flux after the foulant permeation through the membrane, and *J*_0_ is the initial pure water flux.

All the data were collected 3 times, and the average value was used. The obtained average accuracies were as follows: ±0.5% for the rejection coefficient, and ±5% for flux of the porous PVDF and PVDF/nanoparticle membranes.

**Figure 1:**
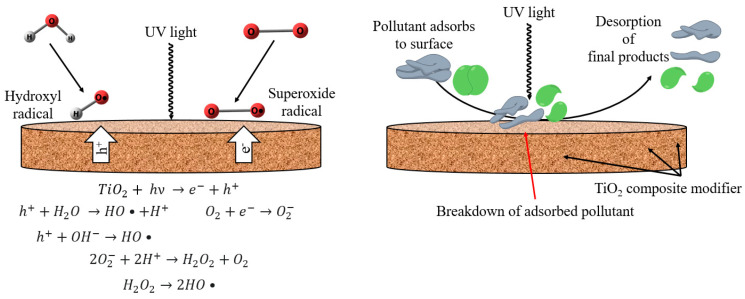
Mechanism of membrane cleaning ability after UV irradiation.

To evaluate the cleaning ability after UV irradiation of porous PVDF membranes after one cycle of BSA filtration and rinsing with water, the membranes in water were immersed under UV lamp irradiation at 230–400 nm with an intensity of 0.10 ± 10%W (DRT-125, MEDtechnique No. 7, St. Petersburg, Russia) for 1–5 h with an interval of 1 h. Then, the membrane was again tested in water ultrafiltration to consider the FRR according to Equation (4) [18]. It was found that after 4 h of UV illumination, the membrane reached its maximum flux recovery ratio, so this time was chosen as optimal. The mechanism of the membrane cleaning ability after UV irradiation is shown in Figure 1.

### 2.13. Atomistic Molecular Dynamics Simulations

Standard values of the OPLS force field [71] were used for MD simulations of PVDF and isopropanol molecules. A chain consisting of 38 monomeric units was used, corresponding to several Kuhn segments for a given polymer. As in our previous studies [22], the potentials for the TiO_2_ molecule, which is a monomer unit of [TiO_2_], were utilized from the work [72]. In the case of the van der Waals interaction of TiO_2_ atoms, the Lennard-Jones potential was adapted by using the Buckingham potential. Water molecules were simulated by the SPCE model [73].

To study the composite PVDF-[TiO_2_], an atomistic MD simulation of 6 different systems was carried out with various combinations, including 240 polymer chains, a [TiO_2_] nanoparticle (consisting of 422 TiO_2_ molecules), and liquid molecules (water and isopropanol). The concentration of [TiO_2_] in the polymer was 4.2 wt%.

The molecular dynamics simulation was carried out by the Gromacs molecular simulation package [74]. Simulations were performed in the NPT ensemble using Berendsen barostat [75] (at 1 atm with the time constant *τ_p_* = 1 ps) and V-rescale thermostat [76] (with the time constant *τ_T_* = 0.4 ps). The simulation step was 2 fs.

For each of the systems, equilibrium was carried out at 500 K for 100 ns. This time was sufficient for mixing and balancing the system. After the system was balanced, it was cooled at a constant rate (2 K/ns) to 300 K. Then, each system was balanced for 1.5 µs at 300 K. At the final stage, simulation trajectories were obtained for 0.5 µs and used for analysis. The density of the system in the absence of nanoparticles and liquid molecules at 300 K is 1.63 ± 0.05 g/cm^3^. This value differs by less than 10% of tabulated data for PVDF (1.78 g/cm^3^). This fact confirms the correctness of the reproduction in the modeling of the polymer system.

## 3. Results and Discussion

Section 3 presents the transport and physicochemical properties of two types of PVDF-based membranes—porous and dense membranes, which were investigated in ultrafiltration and pervaporation, respectively. These membranes were prepared in two different ways (non-solvent induced phase separation (NIPS) for porous membranes and the evaporation induced phase inversion method (EIPS) for dense membranes), so the differences in the structure and mechanism of formation make it possible to evaluate the influence of modifier inclusion (TiO_2_, Ag-TiO_2_, GO-TiO_2_, and MWCNT/TiO_2_) on the transport, structural, and physicochemical properties of the membranes. Additionally, to explain the obtained properties of the membranes, molecular dynamics simulation of PVDF and the TiO_2_ system was carried out, which is also presented in this section.

### 3.1. Development and Investigation of Porous PVDF and PVDF/Nanoparticle Membranes

Porous membranes were fabricated using non-solvent induced phase separation (NIPS). The separation of components in the feed by porous membranes occurs due to the pore size of the membranes and the sieve effect, in which small molecules pass through the membranes while large molecules are retained. The introduction of modifiers (TiO_2_, Ag-TiO_2_, GO-TiO_2_, and MWCNT/TiO_2_) into the PVDF matrix led to a change in the transport and physicochemical characteristics of the membranes.

#### 3.1.1. Ultrafiltration Performance of Porous PVDF and PVDF/Nanoparticle Membranes

**Figure 2:**
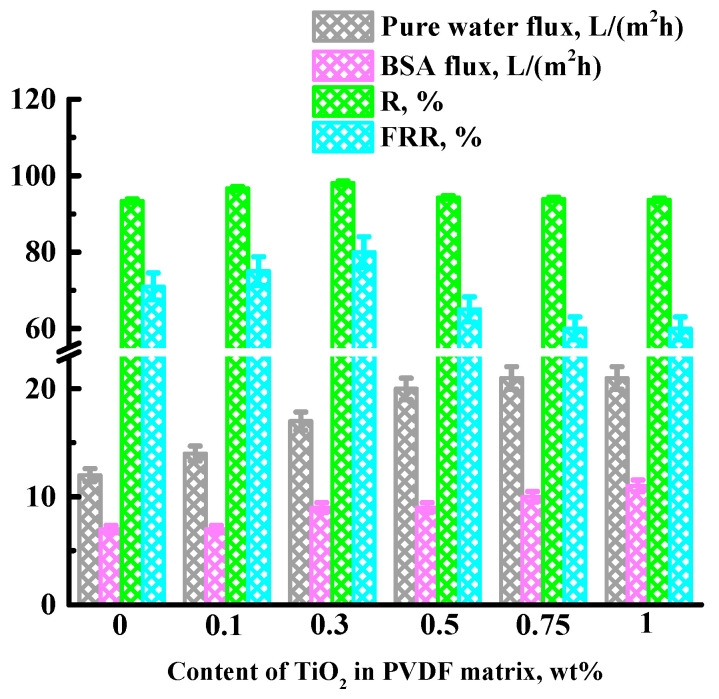
Dependence of pure water flux, BSA flux, BSA rejection coefficient (R), and flux recovery ratio (FRR) on the TiO_2_ concentration in the PVDF matrix during BSA ultrafiltration at 1 bar. The ultrafiltration experiment was carried out over 2 h.

To select the optimal concentration of nanoparticles, 0.1, 0.3, 0.5, 0.75, and 1 wt% TiO_2_ were introduced into the PVDF matrix. The transport properties of the developed porous membranes based on PVDF and its composites with TiO_2_ were studied in ultrafiltration of a water and BSA solution (0.5 wt% in phosphate buffer). The transport properties of porous PVDF and PVDF/nanoparticle membranes are presented in Figure 2.

It was found that the introduction of up to 0.3 wt% TiO_2_ into the PVDF matrix led to an increase in water and BSA fluxes, R and FRR, while a further increase in TiO_2_ concentration led to a further increase in water and BSA fluxes but, at the same time, to a decrease of R and FRR, which may be associated with defects due to TiO_2_ agglomerates. The data on fluxes and the BSA rejection coefficient are in line with the apparent trend that most mixed matrix membranes show higher fluxes and rejection coefficients compared to the pristine porous PVDF membrane [33]. The decrease in flux recovery ratio (FRR) after 0.3 wt% TiO_2_ in the PVDF matrix may be associated with an increase in surface roughness, which could lead to an increase in the specific area of the selective layer for adsorption of BSA. This effect could lead to an increase in surface contamination and a decrease in FRR, which was also noted in [23]. Based on the obtained transport characteristics data, the optimal concentration of TiO_2_ in the PVDF matrix is 0.3 wt%.

**Figure 3:**
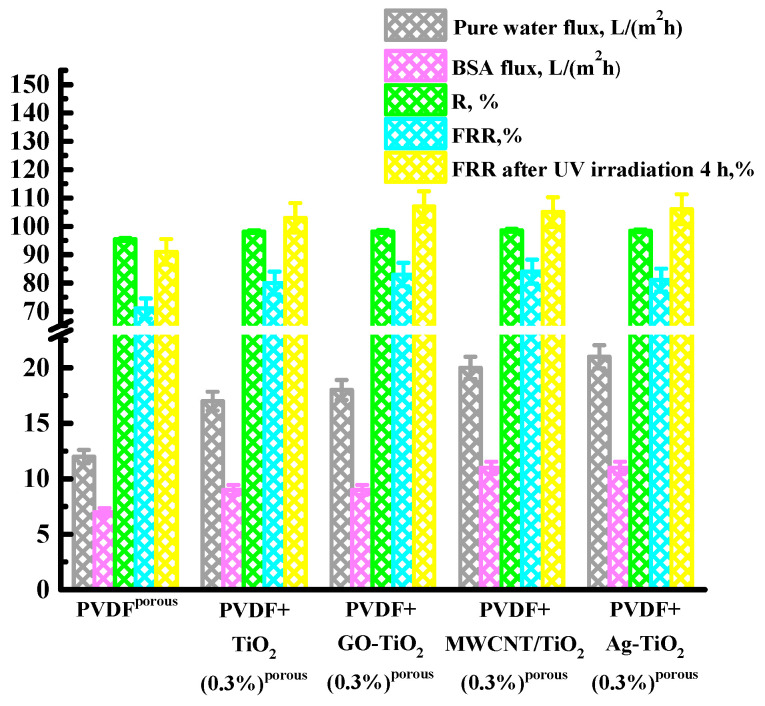
Performance and fouling resistance of porous pristine PVDF and modified PVDF/nanoparticle membranes in ultrafiltration of the BSA solution at 1 bar: water and BSA fluxes, BSA rejection coefficient (R), and flux recovery ratio (FRR). The ultrafiltration experiment was carried out over 7 h.

Next, 0.3 wt% of other nanoparticles (Ag-TiO_2_, GO-TiO_2_, and MWCNT/TiO_2_) were introduced into the PVDF matrix, and their photocatalytic activity was studied. After one cycle of BSA filtration and rinsing with water, the membranes in water were immersed under UV lamp irradiation for 4 h. Then, the pure water flux was measured for 30 min through the membrane to calculate the FRR. The data obtained are presented in Figure 3.

It was found that the introduction of all nanoparticles led to an increase in water and BSA fluxes, the BSA rejection coefficient, and the flux recovery ratio. The PVDF+Ag-TiO_2_(0.3%)^porous^ membrane has the highest flux values for water (21 L/(m^2^h)) and the BSA solution (11 L/(m^2^h)), but, at the same time, the values of the BSA rejection coefficient (98.4%) and flux recovery ratio after washing with water (81%) were a little bit lower compared with the PVDF+MWCNT/TiO_2_(0.3%)^porous^ membrane (water (20 L/(m^2^h)) flux, BSA (11 L/(m^2^h)) flux, BSA rejection coefficient 98.6%, flux recovery ratio after washing with water 84%).

An increase in flux recovery ratio (FRR) after UV irradiation indicates the photocatalytic activity of the developed porous membranes. Membrane surface cleaning occurs due to added nanoparticles (TiO_2_, Ag-TiO_2_, GO-TiO_2_, and MWCNT/TiO_2_), which, upon absorption of a light quantum, generate free charge carriers, such as positive holes and electrons. These free charge carriers, when reacted with water vapor, form oxidants, such as O_2_^−^, -OH, HO_2_^−^, H_2_O_2_, OH‧ radicals that decompose pollutants [22,77]. The mechanism of membrane cleaning ability after UV irradiation is shown in Figure 1. Irradiation of membranes with a UV lamp for 4 h leads to an increase in the flux recovery ratio both for the unmodified PVDF^porous^ membrane and for membranes based on PVDF/nanoparticles. Previously, a FRR of more than 100% was also noted in [16]. For the pristine PVDF^porous^ membrane, an increase in FRR up to 91% was noted. The introduction of 0.3 wt% TiO_2_ led to an increase in FRR up to 103% due to the photocatalytic properties of the modifier. The use of GO-TiO_2_ as a modifier leads to an increase in FRR up to 107%, which is higher than for the membrane modified with TiO_2_. This may be due to the role of GO, which acts as an acceptor of activated electrons from TiO_2_, reducing the carrier recombination and leading to an increase in the photocatalytic efficiency and FRR [33]. The use of MWCNT/TiO_2_ as a modifier leads to an increase in FRR up to 105%, which is higher than for the membrane modified with TiO_2_. This may occur due to the fact that electrons move between MWCNT and TiO_2_ particles, and the presence of MWCNTs can stabilize charge separation and reduce their recombination. In addition, MWCNT particles act as a photosensitizer to improve the photocatalytic properties of titanium dioxide [38]. The use of Ag-TiO_2_ as a modifier leads to an increase in FRR up to 106%, which is also higher than for the membrane modified with TiO_2_. This can be explained by the fact that Ag can absorb photons due to the localized surface plasmon resonance (LSPR) effect and can generate photoelectrons even when irradiated with visible light [78,79]. Further, these photoelectrons are injected into the conduction band of TiO_2_ and can be captured by an oxygen molecule on the surface of TiO_2_. Further reactions occur with the participation of TiO_2_, leading to the formation of free radicals, which increase the degradation and mineralization of pollutants on the membrane surface [18].

Thus, it is found that all developed modified porous membranes possess photocatalytic activity and cleaning ability after UV irradiation. The membranes modified with 0.3 wt% MWCNT/TiO_2_ and Ag-TiO_2_ have the optimal transport properties.

**Figure 4:**
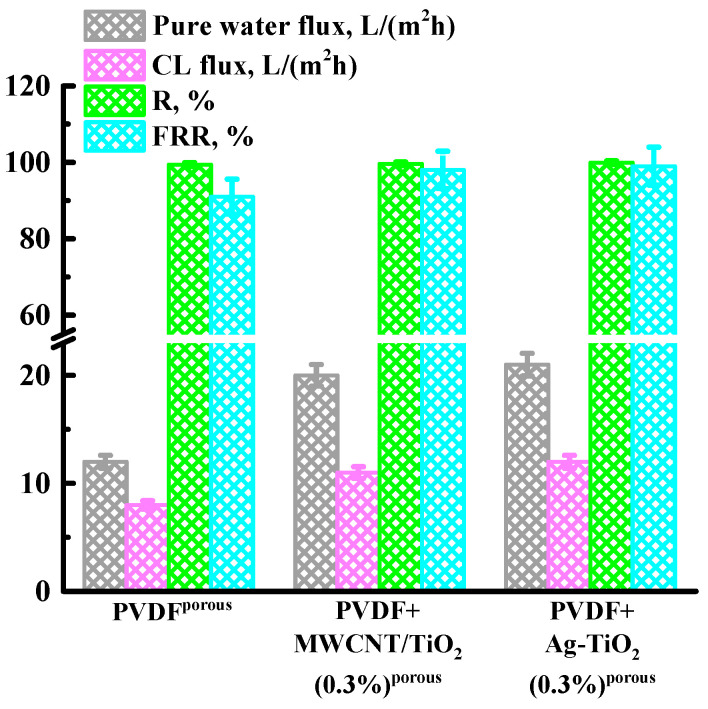
Dependence of pure water flux, CL flux, CL rejection coefficient (R), and flux recovery ratio (FRR) on the TiO_2_ concentration in the PVDF matrix during CL ultrafiltration at 1 bar. The ultrafiltration experiment was carried out for 2 h.

Currently, there is a problem of oil regeneration, utilization of used oils, and wastewater treatment from them. A properly selected ultrafiltration membrane can more effectively solve the problem of water-oil separation compared to traditional separation methods. The transport properties of the initial membrane (PVDF^porous^) and membranes with optimal properties (PVDF+MWCNT/TiO_2_(0.3%)^porous^ and PVDF+Ag-TiO_2_(0.3%)^porous^) were studied in the process of separating a coolant lubricant (cutting fluid) emulsion in water (5 wt% CL in water). The data obtained are presented in Figure 4.

It was found that the introduction of MWCNT/TiO_2_ and Ag-TiO_2_ led to an increase in CL flux, the CL rejection coefficient, and the flux recovery ratio compared to a pristine PVDF^porous^ membrane. For the modified membranes, a high level of FRR 98% for PVDF+MWCNT/TiO_2_(0.3%)^porous^ membrane and 99% for PVDF+Ag-TiO_2_(0.3%)^porous^ membrane was noted after CL, unlike BSA ultrafiltration, where FRR were 84% and 81%, respectively. Therefore, after passing through the membrane CL, the membranes were not exposed to UV irradiation to restore the flux.

#### 3.1.2. Structure and Physicochemical Properties of Porous PVDF and PVDF/Nanoparticle Membranes

To explain the obtained transport characteristics of the developed porous membranes based on PVDF and PVDF/nanoparticle composites, the structure and physicochemical characteristics were studied by SEM and AFM microscopies and contact angle measurements.

**Figure 5:**
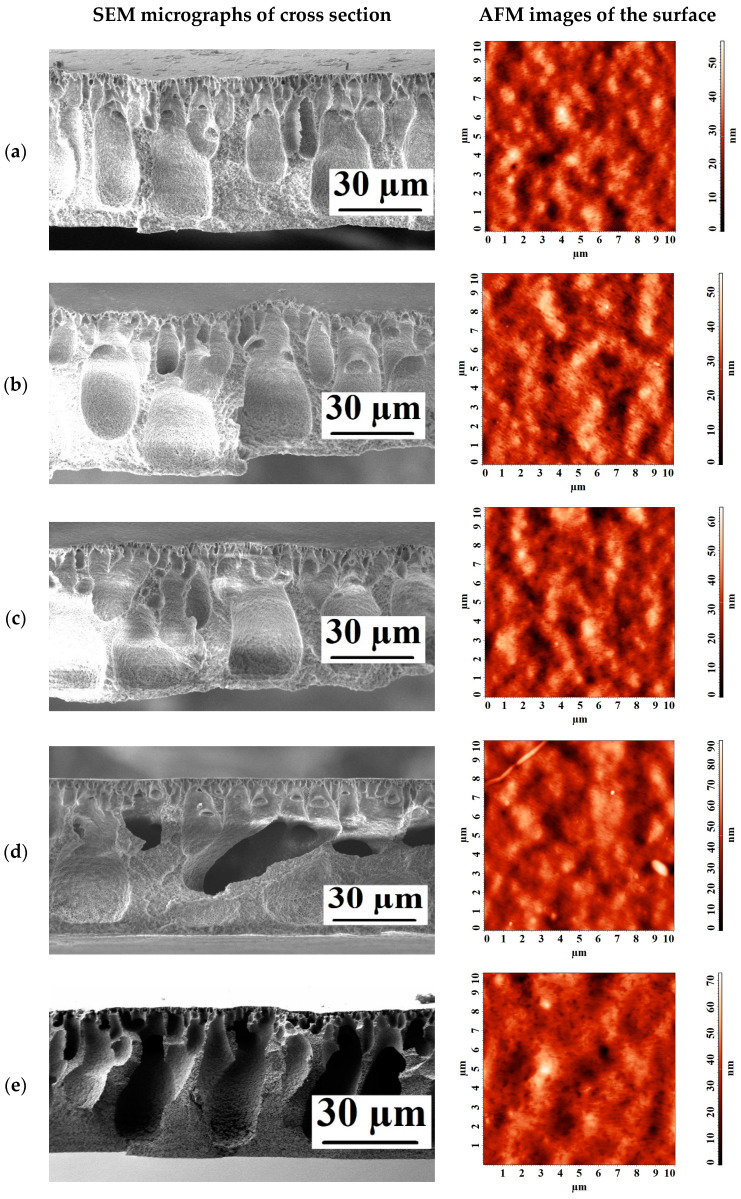
SEM micrographs of the cross section and AFM images of the surface of the developed porous membranes: (**a**) PVDF^porous^, (**b**) PVDF+TiO_2_(0.3%)^porous^, (**c**) PVDF+GO-TiO_2_(0.3%)^porous^, (**d**) PVDF+MWCNT/TiO_2_(0.3%)^porous^, and (**e**) PVDF+Ag-TiO_2_(0.3%)^porous^.

Figure 5 shows the SEM micrographs of the cross section and AFM images of the surface with a scan size of 10 × 10 μm of the developed porous membranes.

It was found that the introduction of nanoparticles led to a slight change in the cross section of porous membranes based on PVDF/nanoparticle composites, compared with the pristine PVDF^porous^ membrane. On SEM micrographs of the cross section, pores in the form of “vacuoles” were noted for all membranes. Based on the AFM images presented in Figure 5, the surface roughness parameters were calculated, which are presented in Table 2. Table 2 also contains the contact angle data studied by the attached bubble method.

It was found that the introduction of nanoparticles did not lead to a significant change in surface roughness (the maximum difference in the roughness parameters is not more than 2.3 nm, which could be the AFM measurement error). Changes in contact angles were noted: contact angles decreased for modified membranes. The decrease in the contact angle for a porous mixed matrix membrane can be attributed to the spontaneous migration of nanoparticles with hydrophilic nature to the polymer/water interface during phase inversion to reduce the interface energy [33,80]. An increase in the hydrophilicity of the membranes is additionally explained by molecular dynamics simulation. This enhanced hydrophilicity led to an increase in the ultrafiltration performance of the membranes.

### 3.2. Development and Investigation of Dense PVDF and PVDF/Nanoparticle Membranes

In contrast to the porous membranes obtained by non-solvent induced phase separation (NIPS), dense membranes were fabricated using the evaporation induced phase inversion method (EIPS). The separation of components by dense membranes occurred due to the “solubility-diffusion” mechanism, in which separation was due to the free volume in the membrane. The introduction of modifiers (TiO_2_, Ag-TiO_2_, GO-TiO_2_, and MWCNT/TiO_2_) into the PVDF matrix led to a change in the transport properties, cross-section morphology, surface roughness, and surface hydrophilicity of the membranes.

#### 3.2.1. Pervaporation Performance of Dense PVDF and PVDF/Nanoparticle Membranes

**Figure 6:**
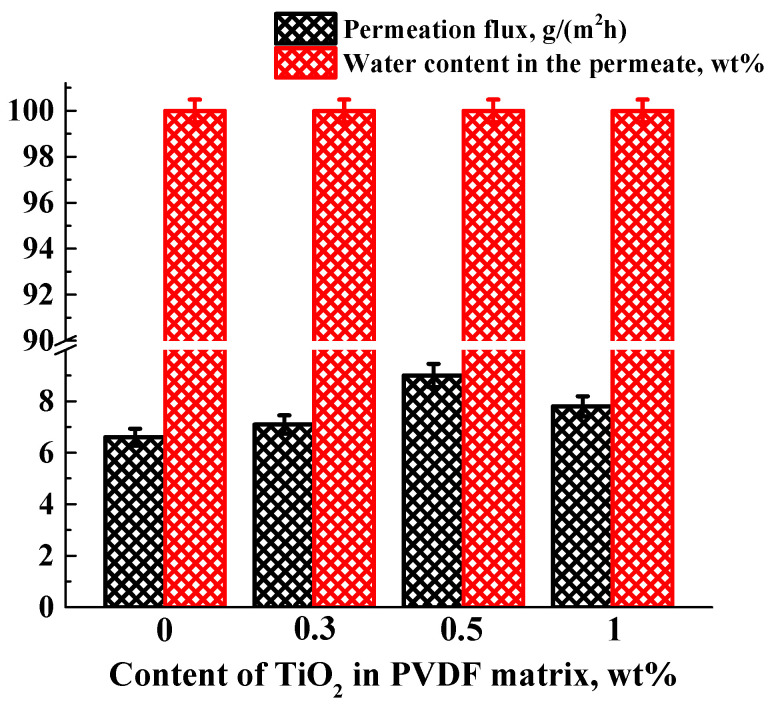
Dependence of the permeation flux and water content in the permeate on the content of TiO_2_ in the PVDF matrix in pervaporation separation of a water/isopropanol (50/50 wt%) mixture at 22 °C.

To select the optimal concentration of nanoparticles, 0.3, 0.5, and 1 wt% TiO_2_ were introduced into the PVDF matrix. The transport properties of the developed dense membranes based on PVDF and its composites with TiO_2_ were studied in pervaporation separation of a water/isopropanol (50/50 wt%) mixture. The dependence of the permeation flux and water content in the permeate on the content of TiO_2_ in the PVDF matrix is shown in Figure 6.

It was found that all dense membranes based on PVDF were highly selective with respect to water (water content in the permeate was 99.99 wt%). Even though PVDF is a hydrophobic polymer, its selectivity to water can be explained by the diffusion selectivity of the membrane. Isopropanol practically did not penetrate the membrane due to its large diameter (~0.47 nm) and the dense structure of the PVDF membrane (high packing density of polymer chains). The same effect for a dense membrane was described in [54], where a water/benzene mixture was separated. The introduction of up to 0.5 wt% TiO_2_ led to an increase in permeation flux from 6.6 g/(m^2^h) for an unmodified PVDF^dense^ membrane to 9 g/(m^2^h) for a PVDF+TiO_2_(0.5%)^dense^ membrane during separation of a water/isopropanol (50/50 wt%) mixture. A further increase in the TiO_2_ content in the PVDF matrix led to a decrease in the permeation flux, which may be due to TiO_2_ agglomeration. The introduction of titanium dioxide led to the hydrophilization of the membranes confirmed by the measurement of the contact angles (contact angle data presented below) and molecular dynamics simulation, resulting in an increase in the permeation flux of the membranes. Thus, the concentration of 0.5 wt% was chosen as optimal for PVDF membranes, and 0.5 wt% of the other investigated nanoparticles (Ag-TiO_2_, GO-TiO_2_, and MWCNT/TiO_2_) were introduced into the PVDF matrix.

**Figure 7:**
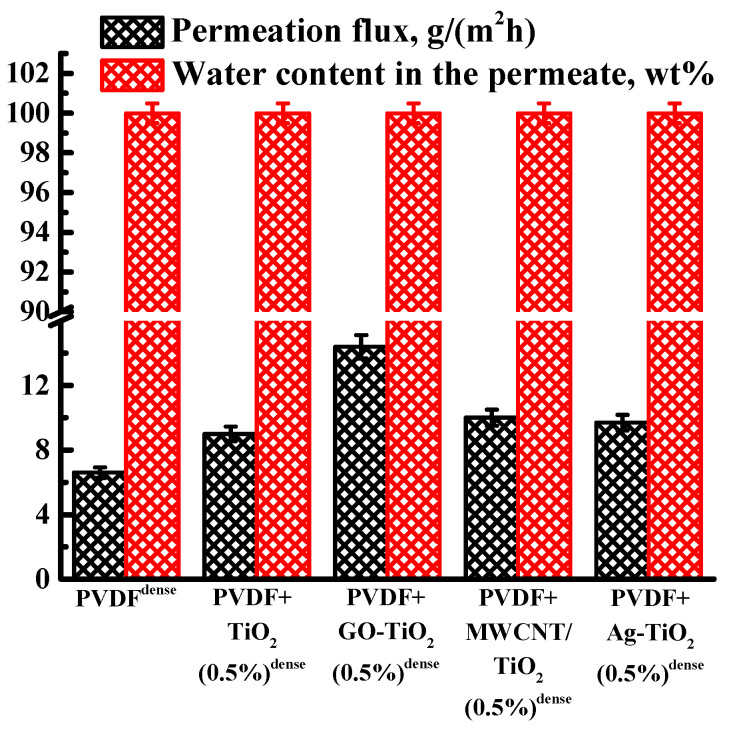
Permeation flux and water content in the permeate for dense membranes-based PVDF and PVDF/nanoparticle composites in pervaporation separation of a water/isopropanol (50/50 wt%) mixture at 22 °C.

The transport properties of the developed dense membranes based on PVDF and its composites with nanoparticles (Ag-TiO_2_, GO-TiO_2_, and MWCNT/TiO_2_) were studied in pervaporation separation of a water/isopropanol (50/50 wt%) mixture. The permeation flux and water content in the permeate for dense membranes-based PVDF/nanoparticles are shown in Figure 7 (the data for PVDF^dense^ and PVDF+TiO_2_(0.5%)^dense^ membranes are also presented in Figure 7 for comparison).

It was found that all dense PVDF-based membranes are highly selective with respect to water; the water content in the permeate for all membranes was 99.99 wt%. The introduction of 0.5 wt% modified nanoparticles led to a greater increase in the permeation flux in pervaporation separation of a water/isopropanol (50/50 wt%) mixture. The highest increase in the permeation flux (ca. 2.2 times higher compared with a pristine membrane) was noted for the PVDF+GO-TiO_2_(0.5%)^dense^ membrane. It can be explained by the highest values of the surface roughness parameters (AFM data presented below).

#### 3.2.2. Structure and Physicochemical Properties of Dense PVDF and PVDF/Nanoparticle Membranes

To explain the obtained transport characteristics, the structural and physicochemical properties of the dense membranes were studied by scanning electron and atomic force microscopies, FTIR spectroscopy, TGA, and measurement of contact angles.

***Fourier transform infrared spectroscopy***

**Figure 8:**
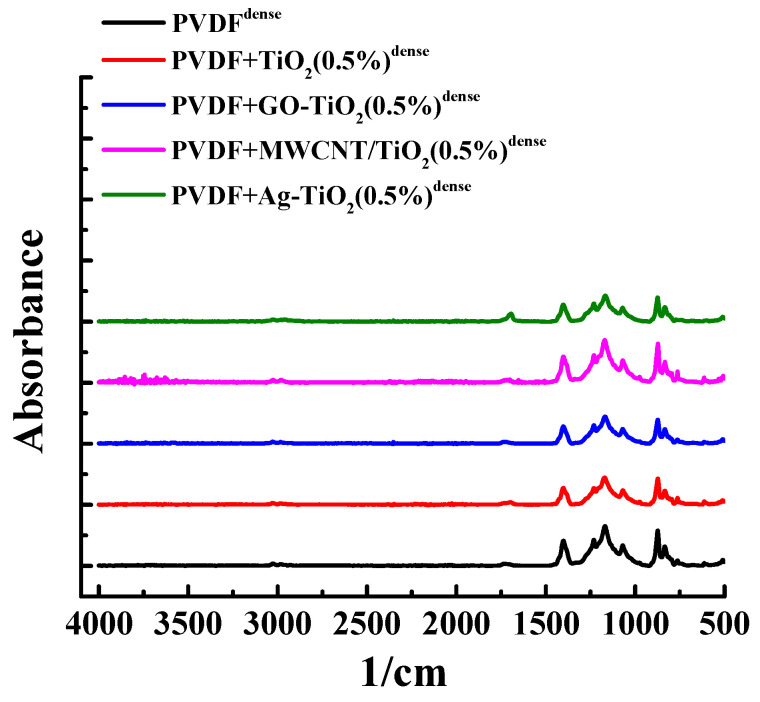
FTIR spectra for dense membranes based on PVDF and its composites with nanoparticles.

Structural changes in dense membranes based on PVDF and its composites with nanoparticles were studied using Fourier transform infrared spectroscopy (FTIR). FTIR spectra are shown in Figure 8.

The FTIR spectrum of the PVDF membrane showed characteristic peaks for this polymer: at 2983 and 2928 cm^−1^ peaks associated with asymmetric stretching of -CH_2_ groups, peaks at 763, 834 cm^−1^ associated with vibrations of -CH_2_ groups and asymmetric stretching of -CF_2_ groups, the peak at 510 cm^−1^ associated with the bending of -CF_2_ groups, and at 480 cm^−1^ the bending and vibration of -CF_2_ groups [81]. The introduction of 0.5 wt% nanoparticles (TiO_2_, Ag-TiO_2_, GO-TiO_2_, and MWCNT/TiO_2_) into the PVDF matrix did not lead to strong changes in the FTIR spectra, which may be due to low modifier concentration [82].

***Scanning electron microscopy***

**Figure 9:**
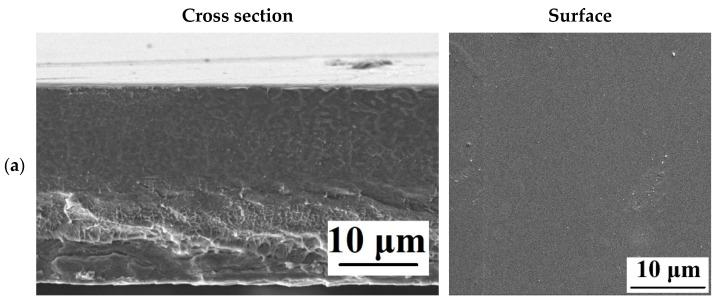

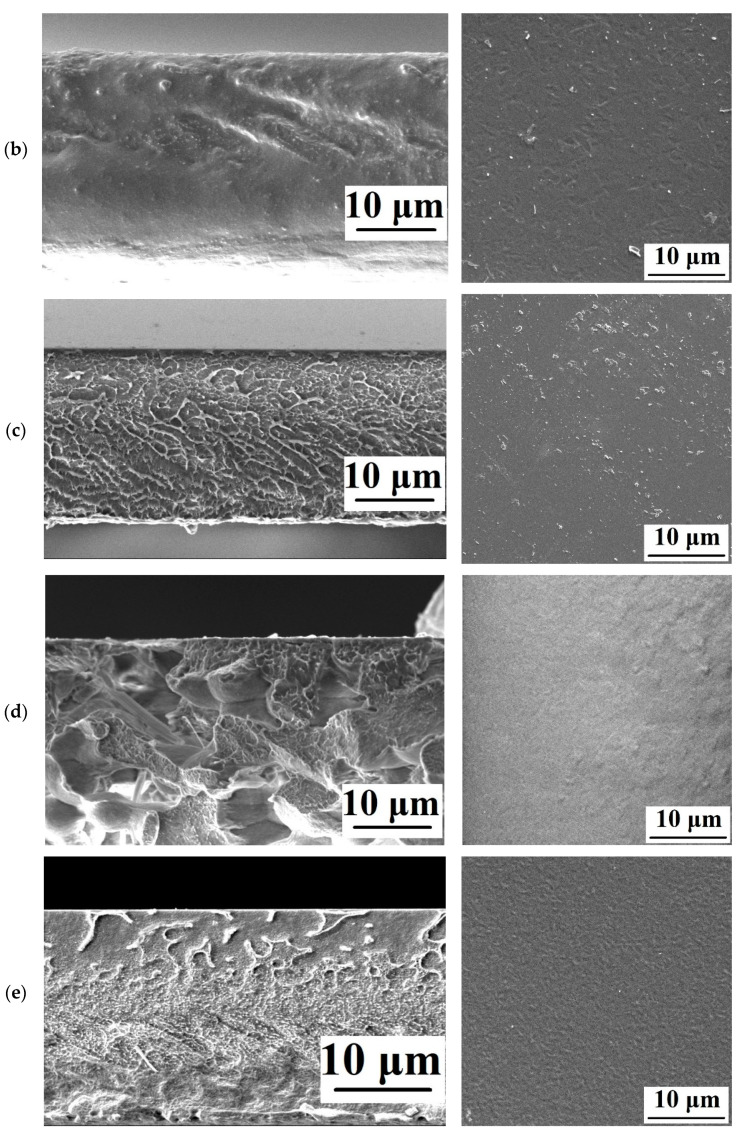
SEM micrographs of the cross section and surface of the developed dense membranes: (**a**) PVDF^dense^, (**b**) PVDF+TiO_2_(0.5%)^dense^, (**c**) PVDF+GO-TiO_2_(0.5%)^dense^, (**d**) PVDF+MWCNT/TiO_2_(0.5%)^dense^, and (**e**) PVDF+Ag-TiO_2_(0.5%)^dense^.

The morphology of the developed dense membranes based on PVDF and its composites with nanoparticles was studied by scanning electron microscopy (SEM). SEM micrographs of the cross section and surface of the developed pervaporation dense membranes based on PVDF and its composites with nanoparticles are shown in Figure 9.

When nanoparticles were introduced into the PVDF matrix, significant changes occurred in the cross section of dense membranes, which became more ribbed and wavier. The surface of the developed membranes changed less significantly, but SEM micrographs of the surface showed modifier nanoparticles, especially in the case of the PVDF+GO-TiO_2_(0.5%)^dense^ membrane, which had the highest surface roughness (AFM data presented below) and permeation flux (Figure 7).

***Atomic force microscopy***

**Figure 10:**
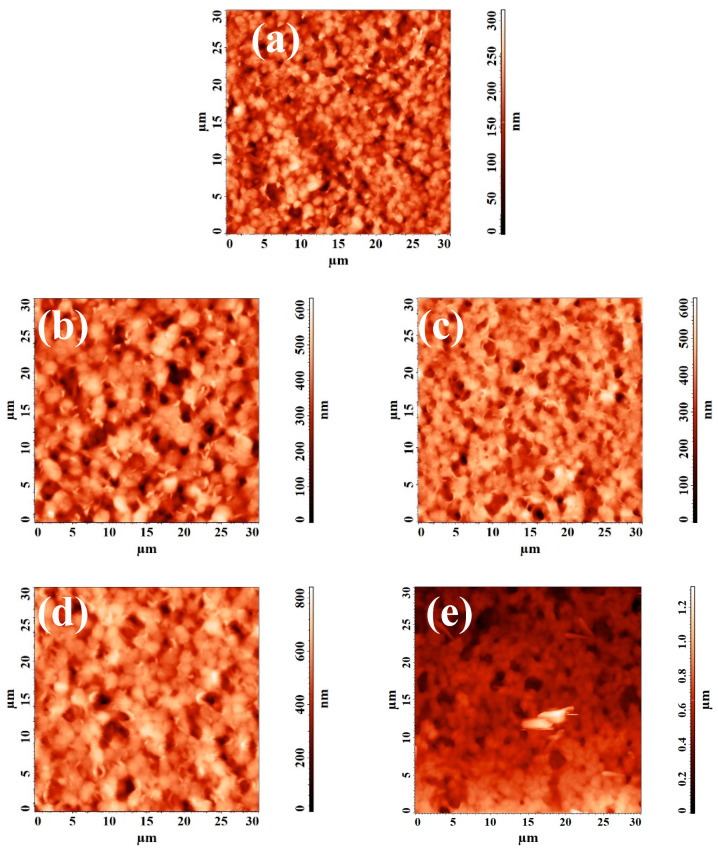
AFM images of the developed dense membranes: (**a**) PVDF^dense^, (**b**) PVDF+TiO_2_(0.5%)^dense^, (**c**) PVDF+GO-TiO_2_(0.5%)^dense^, (**d**) PVDF+MWCNT/TiO_2_(0.5%)^dense^, and (**e**) PVDF+Ag-TiO_2_(0.5%)^dense^.

The surface topology of membranes based on PVDF and its composites with nanoparticles was studied by atomic force microscopy (AFM). AFM images of the surface with a scan size of 30 × 30 µm are shown in Figure 10.

Based on the obtained AFM images, the characteristics of the membrane surface roughness (root-mean-squared (Rq) and average roughness (Ra)) were calculated and presented in Table 3.

It was found that the introduction of nanoparticles into the PVDF matrix led to an increase in the surface roughness parameters, causing an increase in the permeation flux in the pervaporation separation of a water/isopropanol mixture (Figure 7). The highest values of the roughness parameters were noted for the membrane modified with 0.5 wt% GO-TiO_2_ (PVDF+GO-TiO_2_(0.5%)^dense^ membrane), which was confirmed by surface SEM micrographs (Figure 9) and corresponded to the maximum values of the permeation flux (Figure 7).

***Contact angle***

To study changes in the hydrophilic/hydrophobic properties of the surface of the developed pervaporation dense membranes based on PVDF and its composites with nanoparticles, the contact angles of water and glycerol were measured. Critical surface tension dispersion and polar components were also calculated. The data are presented in Table 4.

It has been found that the contact angles of water for modified membranes were reduced compared to the PVDF^dense^ membrane due to the hydrophilic nature of modifiers. Depending on the modifier, the contact angle of water decreased by 5–9 degrees. The contact angles of glycerol were close for all developed membranes. At the same time, the introduction of TiO_2_, MWCNT/TiO_2_, and Ag-TiO_2_ led to a slight increase in the contact angles of glycerol, and GO-TiO_2_ to a decrease in comparison with the initial PVDF^dense^ membrane. The PVDF+GO-TiO_2_(0.5%)^dense^ membrane had the smallest contact angle of water (most hydrophilic surface) and glycerol, which may be due to the presence of functional oxygen-containing groups in the GO structure [83]. It also determined the highest permeation flux of this membrane.

The contact angle of two liquids (water and glycerol) were used to calculate the critical surface tension of dense membranes. The polar ($\sigma_{s}^{p}$) and dispersion ($\sigma_{s}^{d}$) components of the surface tension were calculated separately, characterizing the polar and dispersion interactions between the membrane and the studied liquids. According to the data of Table 4, the dispersion component decreased for membranes modified with nanoparticles, and the polar component increased. The results obtained indicate an increase in the hydrophilic properties of the surface of polymeric membranes as a result of their modification with nanoparticles.

***Thermogravimetric analysis***

**Figure 11:**
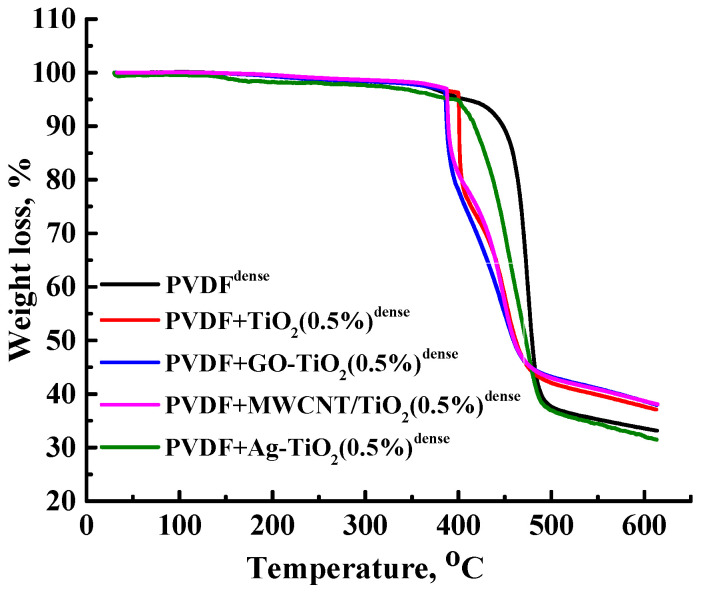
TG curves for dense membranes based on PVDF and its composites with nanoparticles.

The thermal stability of the dense membranes based on PVDF and its composites with nanoparticles was investigated by thermogravimetric analysis (TGA). The obtained thermograms (TG) are presented in Figure 11.

A three-step weight loss was observed for membranes based on PVDF and its composites with nanoparticles. The first visible weight loss of membranes occurred up to 385 °C and can be attributed to the elimination of residual solvent (DMA) and moisture. The total weight loss in the first stage was nearly ∼3–6%. The pristine dense PVDF membrane had an initial degradation temperature from 375 to 425 °C. The degradation temperature of the dense membranes based on PVDF/nanoparticle composites was lower than that of the pristine dense PVDF membrane that may be related to the catalytic effect of TiO_2_ on the decomposition of PVDF, which was also noted in [84]. The second weight loss occurred between 385 and 500 °C, owing to the primary degradation of the PVDF [85]. The third weight loss, owing to the second stage of degradation, proceeded from 490 °C and was manifested by a slight change in slope compared to the first stage of degradation (from 385 to 490 °C), in which the bulk of the polymer mass was lost [85]. PVDF+TiO_2_(0.5%)^dense^, PVDF+GO-TiO_2_(0.5%)^dense^, PVDF+MWCNT/TiO_2_(0.5%)^dense^ membranes showed less weight loss compared to PVDF^dense^ and PVDF+Ag-TiO_2_(0.5%)^dense^ at 600 °C. As can be seen from the results obtained, these additives contributed to the stabilization of PVDF during thermal decomposition. The data obtained indicate the possibility of using the developed membranes at elevated temperatures to 350 °C.

Thus, it was found that when porous membranes were prepared by non-solvent induced phase separation (NIPS), the maximum concentration of modifiers was 0.3 wt%, while for pervaporation membranes prepared by the evaporation induced phase inversion method (EIPS), it was 0.5 wt%. This confirms that the concentration of the modifier depends not only on its type but also on the method of preparation of mixed matrix membranes. However, for both membrane types, the inclusion of a modifier caused the enhanced transport performance due to changes in the structural and physicochemical properties of developed membranes.

### 3.3. Comparison of the Performance with Membranes

The comparison of the ultrafiltration performance of the porous PVDF+MWCNT/TiO_2_(0.3%)^porous^ and PVDF+Ag-TiO_2_(0.3%)^porous^ membranes to the porous PVDF-based membranes described in the literature for the ultrafiltration of BSA under close experimental conditions is presented in Table 5.

It was demonstrated that the developed porous PVDF+MWCNT/TiO_2_(0.3%)^porous^ and PVDF+Ag-TiO_2_(0.3%)^porous^ membranes had good membrane performance in the ultrafiltration of the BSA solution, a high level of BSA rejection coefficients, and FRR after UV-irradiation. This demonstrated the promising application of the developed porous membranes in the industrial ultrafiltration separation.

### 3.4. Molecular Dynamics Simulation of PVDF and TiO_2_ System

To explain the increase in hydrophilicity and permeability of the hydrophobic PVDF membrane after the introduction of the modifier, a molecular dynamics (MD) simulation was applied.

From the simulation data, it was found that in a system without a [TiO_2_] nanoparticle, water molecules are assembled into clusters (up to 10–50 molecules). This is due to the fact that water molecules do not form hydrogen bonds with the polymer but interact with each other and with isopropanol (IPA) molecules. To illustrate this fact, the number of hydrogen bonds H_2_O–H_2_O and IPA–H_2_O in Figure 10 (red columns) is given. The result obtained indicates the hydrophobic properties of the polymer, which are consistent with both the literature data [60] and the results obtained in this study.

**Figure 12:**
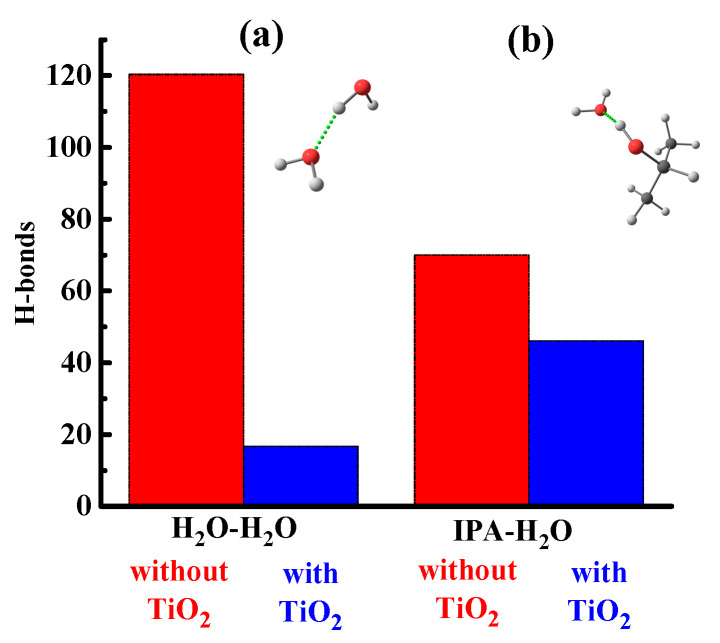
Number of hydrogen bonds between (**a**) water molecules and (**b**) water and isopropanol molecules per 100 solvent molecules.

**Figure 13:**
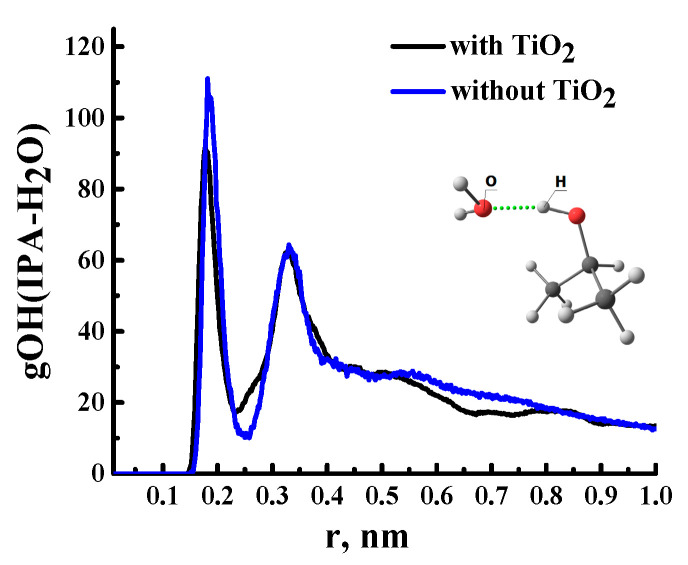
Pair radial distribution function for O and H atoms of IPA and H_2_O molecules, respectively.

**Figure 14:**
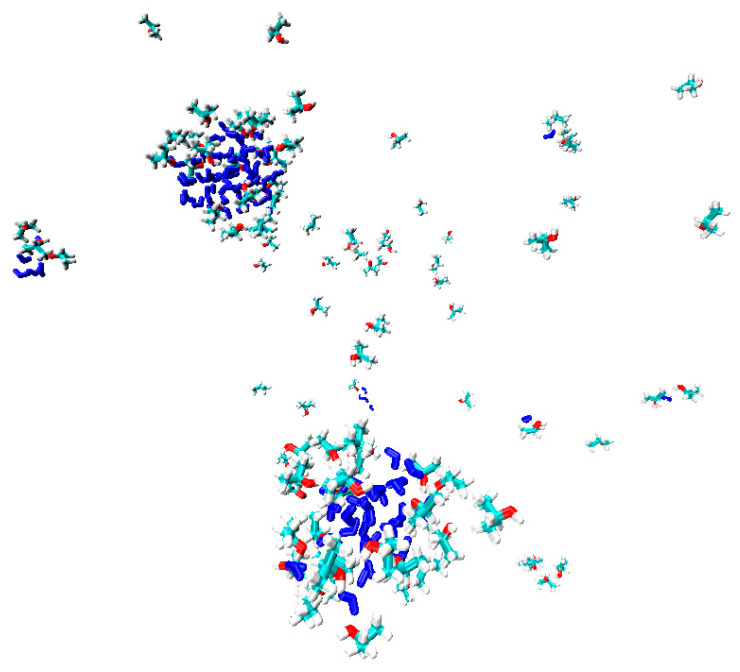
Snapshot of the PVDF/IPA+H_2_O system. PVDF atoms are hidden for clarity.

A different situation is observed for the distribution of IPA molecules in the polymer matrix, which are not characterized by the formation of large clusters. Solvent molecules are distributed in the bulk of the polymer, mainly in a single state, or are sorbed on the surface of water clusters. Sorption on the surface of the water cluster is due to the formation of hydrogen bonds between IPA and H_2_O (Figure 12). Additionally, the presence of hydrogen bonds between the molecules of water and isopropanol is confirmed by the pair distribution function for oxygen atoms of IPA and hydrogen H_2_O (Figure 13). For an illustration, Figure 14 shows water clusters (blue) in the snapshot of the system without a nanoparticle, as well as the sorption of isopropanol molecules on the surface of the water cluster.

**Figure 15:**
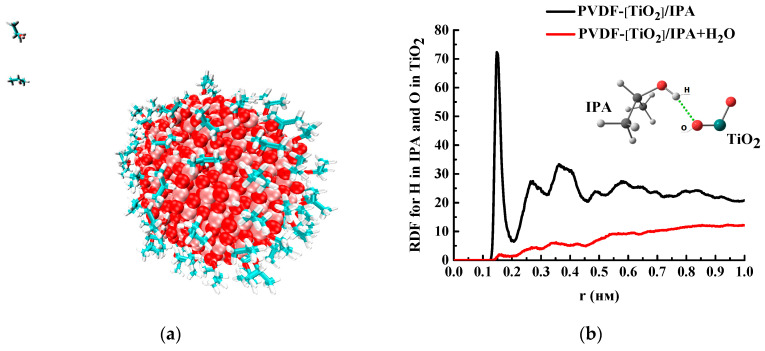
(**a**) Snapshot of the PVDF-[TiO_2_]/IPA system (PVDF atoms are hidden for clarity). (**b**) Pair radial distribution function for O and H atoms of TiO_2_ and water molecules, respectively.

**Figure 16:**
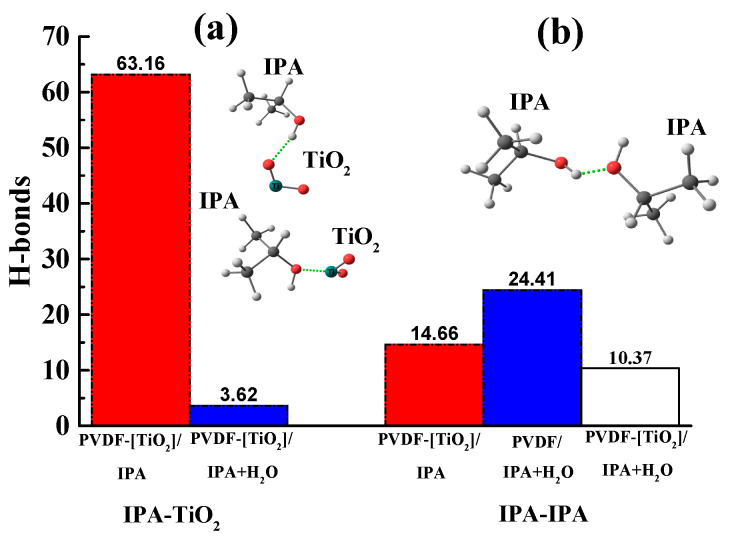
Number of hydrogen bonds between (**a**) isopropanol and titanium dioxide and (**b**) water and isopropanol molecules per 100 solvent molecules.

In the PVDF-[TiO_2_] system with the addition of IPA molecules, but in the absence of water, almost all of the isopropanol is sorbed onto the nanoparticle (Figure 15a). To confirm this fact, the pair distribution functions of the IPA and nanoparticle atoms are shown in Figure 15b, where for the system in the absence of water, a peak is observed at 0.15 nm. This peak is due to the presence of a hydrogen bond between [TiO_2_] and IPA (Figure 16). Moreover, the hydrogen bond between the nanoparticle and IPA (average length d = 0.15 nm) is more energetically favorable than IPA–IPA (d = 0.18 nm). Apparently, this is what determines the sorption of IPA on the nanoparticle surface in this system.

**Figure 17:**
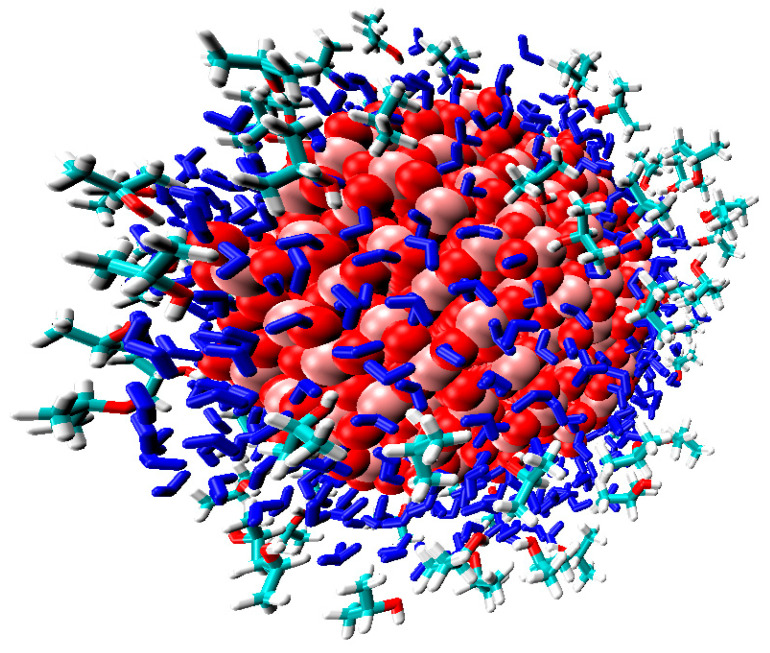
Instant configuration of the PVDF-[TiO_2_]/IPA+H_2_O system.

When water is added to the system considered above and MD simulation is continued, the IPA molecules in the solvate shell of the [TiO_2_] nanoparticle are replaced by water molecules (Figure 17). This is because hydrogen bonds between water and TiO_2_ are more favorable than between IPA and TiO_2_ since IPA-TiO_2_ hydrogen bonds practically disappear (reduce from 63.2 to 3.6 per 100 solvent molecules) (Figure 16).

Thus, it can be concluded that the addition of a nanoparticle to the polymer matrix will lead to an increase in the hydrophilicity of the system, and, consequently, it will increase the permeability of water through the polymer membrane. This result is in qualitative agreement with the results of transport characteristics in the pervaporation (Figure 6) and ultrafiltration (Figure 2).

## 4. Conclusions

In the present study, the influence of various modifiers possessed photocatalytic properties (TiO_2_, Ag-TiO_2_, GO-TiO_2_, and MWCNT/TiO_2_), and the method of membrane preparation (non-solvent induced phase separation (NIPS) for porous membranes and the evaporation induced phase inversion method (EIPS) for dense membranes) on the transport, structural, and physicochemical characteristics of membranes were studied.

Porous membranes were developed for purification of pollutants from aqua solutions in ultrafiltration using the BSA solution as an example. The introduction of 0.3 wt% of modifiers led to an improvement in the transport characteristics of porous membranes compared to the pristine membrane, namely, to enhanced water and BSA fluxes, the BSA rejection coefficient, the flux recovery ratio, and the cleaning ability under UV illumination. Dense membranes were developed for pervaporation separation of a water/isopropanol mixture. The introduction of 0.5 wt% of modifiers led to an increase in permeation flux maintaining high selectivity with respect to water (99.99 wt% water in the permeate). The transport performance changes were attributed to the surface hydrophilization of porous modified membranes (proven with contact angle measurement) and changed cross-sectional structure, increased surface roughness, and decreased contact angles for dense modified membranes (proven with SEM, AFM, and contact angle measurement). Among porous membranes, optimal transport properties have been obtained for membranes modified with 0.3 wt% MWCNT/TiO_2_ and Ag-TiO_2_ based on ultrafiltration separation of the BSA solution. A dense membrane modified with 0.5 wt% GO-TiO_2_ had the optimal properties based on pervaporation separation of a water/isopropanol (50/50 wt%) mixture. Additionally, molecular dynamics simulation of PVDF and the TiO_2_ system demonstrated that the addition of a modifier to the polymer matrix led to an increase in the hydrophilicity of the system and, as a consequence, to the increase in the permeability of water through the polymer membrane that was confirmed by data obtained in pervaporation in ultrafiltration.

## Figures and Tables

**Table 1:** Developed dense and porous membranes based on PVDF and PVDF/nanoparticles composites.

Membrane	Type of Membrane	Type of Nanoparticles	Content of Nanoparticles, wt%
PDVF^porous^	porous	-	-
PDVF+TiO_2_(0.1)^porous^	porous	TiO_2_	0.1
PDVF+TiO_2_(0.3)^porous^	porous	TiO_2_	0.3
PDVF+TiO_2_(0.5)^porous^	porous	TiO_2_	0.5
PDVF+TiO_2_(0.75)^porous^	porous	TiO_2_	0.75
PDVF+TiO_2_(1)^porous^	porous	TiO_2_	1
PVDF+GO-TiO_2_(0.3%)^porous^	porous	GO-TiO_2_	0.3
PVDF+MWCNT/TiO_2_(0.3%)^porous^	porous	MWCNT/TiO_2_	0.3
PVDF+Ag-TiO_2_(0.3%)^porous^	porous	Ag-TiO_2_	0.3
PDVF^dense^	dense	-	-
PDVF+TiO_2_(0.3)^dense^	dense	TiO_2_	0.3
PDVF+TiO_2_(0.5)^dense^	dense	TiO_2_	0.5
PDVF+TiO_2_(1)^dense^	dense	TiO_2_	1
PVDF+GO-TiO_2_(0.5%)^dense^	dense	GO-TiO_2_	0.5
PVDF+MWCNT/TiO_2_(0.5%)^dense^	dense	MWCNT/TiO_2_	0.5
PVDF+Ag-TiO_2_(0.5%)^dense^	dense	Ag-TiO_2_	0.5

**Table 2:** Average (Ra) and root-mean-squared (Rq) roughness values and contact angles for porous PVDF and PVDF/nanoparticle membranes.

Membrane	Ra, nm	Rq, nm	Contact Angle, °
PVDF ^porous^	5.7	7.2	27
PVDF+TiO_2_(0.3%)^porous^	6.0	7.5	24
PVDF+GO-TiO_2_(0.3%)^porous^	6.8	8.5	20
PVDF+MWCNT/TiO_2_(0.3%)^porous^	7.5	9.5	20
PVDF+Ag-TiO_2_(0.3%)^porous^	6.2	8.0	23

**Table 3:** Roughness parameters of dense membranes based on PVDF and its composites with nanoparticles.

Membrane	Ra, nm	Rq, nm
PVDF^dense^	21.1	27.0
PVDF+TiO_2_(0.5%)^dense^	52.0	65.1
PVDF+GO-TiO_2_(0.5%)^dense^	76.8	99.1
PVDF+MWCNT/TiO_2_(0.5%)^dense^	74.2	96.3
PVDF+Ag-TiO_2_(0.5%)^dense^	64.9	87.2

**Table 4:** Contact angles and critical surface tension for dense membranes based on PVDF and its composites with nanoparticles.

Membrane	Contact Angle, °		Critical Surface Tension		
	Water	Glycerol	$\sigma_{s}^{d}$	$\sigma_{s}^{p}$	$\sigma_{s}$
PVDF^dense^	82	81	4.66	19.32	23.98
PVDF+TiO_2_(0.5%)^dense^	74	83	0.00	42.46	42.46
PVDF+GO-TiO_2_(0.5%)^dense^	73	79	0.55	37.24	37.79
PVDF+MWCNT/TiO_2_(0.5%)^dense^	76	83	0.16	36.86	37.01
PVDF+Ag-TiO_2_(0.5%)^dense^	75	83	0.03	39.62	39.65

**Table 5:** Ultrafiltration performance of the porous PVDF+MWCNT/TiO_2_(0.3%)^porous^ and PVDF+Ag-TiO_2_(0.3%)^porous^ membranes and the PVDF-based membranes described in the literature.

Membranes	Pure Water Flux, L/(m^2^h)	BSA Flux, L/(m^2^h)	FRR, %	FRR after UV-Irradiation, %	Rejection Coefficient, %	References
PVDF+MWCNT/TiO_2_(0.3%)^porous^	20	11	84	105	98.6	This study
PVDF+Ag-TiO_2_(0.3%)^porous^	21	11	81	106	98.4	This study
PVDF+Ag-TiO_2_(0.06%)	~90	~10	-	~100	89.8	[18]
PVDF+TiO_2_(30%)	~90	29	103	112	-	[16]
PVDF+GO(2%)	27	11	87	-	-	[86]
PVDF+GO/TiO_2_	488	~325	71	82	92.5	[33]
PVDF+TiO_2_(20%)	~100	~50	60.2	97	85.6	[87]
PVDF+MIL-53(Al)(5%)	44	~25	89	-	80.3	[88]
PVDF-g-PAA (polyvinylidene fluoride-g-polyacrylic acid)	~160	~60	~80	-	~83	[89]

## Data Availability

Not applicable.

## List of Symbols and Abbreviations

AFM	atomic force microscopy
BSA	bovine serum albumin
CL	coolant lubricant
DMA	N,N′-dimethylacetamide
EIPS	evaporation induced phase inversion method
FTIR	Fourier transform infrared spectroscopy
GO	graphene oxide
i-PrOH	isopropanol
LSPR	localized surface plasmon resonance
MD	molecular dynamics
MWCNT	multi-walled nanotubes
NIPS	non-solvent induced phase separation
PA	poly(m-phenylene isophtalamide)
PAA	polyacrylic acid
PAIU	polyamidoimideurea
PAN	polyacrylonitrile
PES	polyethersulfone
PSF	polysulfone
PVDF	poly(vinylidene fluoride)
PVP K-30	polyvinylpyrrolidone K-30
SEM	scanning electron microscopy
TGA	thermogravimetric analysis
TiO_2_	titanium dioxide
UV	ultraviolet
FRR	flux recovery ratio, %
J	flux, L/(m^2^h)
Q	permeation flux, kg/(m^2^h)
R	rejection coefficient, %
